# Extracellular vesicles would be involved in the release and delivery of seminal TGF-β isoforms in pigs

**DOI:** 10.3389/fvets.2023.1102049

**Published:** 2023-02-10

**Authors:** Lorena Padilla, Isabel Barranco, Jesús Martínez-Hernández, Ana Parra, Inmaculada Parrilla, Luis Miguel Pastor, Heriberto Rodriguez-Martinez, Xiomara Lucas, Jordi Roca

**Affiliations:** ^1^Biotechnology of Animal and Human Reproduction (TechnoSperm), Department of Biology, Faculty of Sciences, Institute of Food and Agricultural Technology, University of Girona, Girona, Spain; ^2^Department of Medicine and Animal Surgery, Faculty of Veterinary Science, University of Murcia, Murcia, Spain; ^3^Department of Veterinary Medical Sciences, University of Bologna, Bologna, Italy; ^4^IMIB-Arrixaca, Regional Campus of International Excellence, University of Murcia, Murcia, Spain; ^5^Department of Cell Biology and Histology, School of Medicine, University of Murcia, Murcia, Spain; ^6^Department of Biomedical and Clinical Sciences (BKV), Linköping University, Linköping, Sweden

**Keywords:** extracellular vesicles, porcine, semen, seminal plasma, sperm, TGF-β isoforms

## Abstract

**Introduction:**

Pig seminal plasma (SP) is rich in active forms of all three isoforms (1-3) of transforming growth factor β (TGF-β), a chemokine modulatory of the immune environment in the female genital tract once semen is delivered during mating or artificial insemination (AI). The present study aimed to examine how TGF-βs are secreted by the epithelium of the male reproductive tract and how they are transported in semen, emphasizing the interplay with seminal extracellular vesicles (sEVs).

**Methods:**

Source of TGF-βs was examined by immunohistochemistry in testis, epididymis, and accessory sex glands, by immunocytochemistry in ejaculated spermatozoa, and by Luminex xMAP^®^ technology in SP and sEVs retrieved from healthy, fertile male pigs used as breeders in AI programs.

**Results:**

All three TGF-β isoforms were expressed in all reproductive tissues explored and would be released into ductal lumen either in soluble form or associated with sEVs. Ejaculated spermatozoa expressed all three TGF-β isoforms, both inside and outside, probably the outer one associated with membrane-bound sEVs. The results confirmed that pig SP contains all three TGF-β isoforms and demonstrated that a substantial portion of them is associated with sEVs.

**Discussion:**

Seminal EVs would be involved in the cellular secretion of the active forms of seminal TGF-β isoforms and in their safe transport from the male to the female reproductive tract.

## 1. Introduction

Seminal plasma (SP) is endowed with a plethora of signaling agents involved in the establishment of a state of immune ‘tolerance' in the female genital tract, essential for the successful development and implantation of embryos and subsequent placentation considering their hemi-allogeneic status (1). Cytokines, a family of low-molecular weight proteins secreted mainly by immune cells but also by male genital epithelia and endothelial cells, are considered the major seminal bioactive drivers for this maternal immune tolerance (1–3).

A wide repertoire of cytokines has been identified in SP from several mammalian species (4–7), including porcine (8). Among them is the transforming growth factor beta (TGF-β), a modulatory, mostly anti-inflammatory pleiotropic polypeptide found in mammals in three different isoforms, the TGF-β1, -β2, and -β3 (9). The pig SP is rich in cytokines, with abundance of TGF-β isoforms (5, 8, 10, 11). Seminal TGF-βs are among the SP molecules promoting maternal tolerance to paternal antigens (12–14) as well as initiating a cascade of molecular and cellular events preceding embryo implantation (15). Seminal TGF-βs also increase the expression of endometrial inflammatory mediators, such as interleukin-6 and granulocyte-macrophage colony stimulating factor (16), which facilitate embryo development as demonstrated in several mammalian species (17–21).

Some tissues of the male reproductive tract are known to express TGF-β isoforms and secrete them into the extracellular milieu (22, 23). However, little is known about how this secretion of TGF-β isoforms occurs and how they are transported in semen so that they can remain functionally active in the female genital tract to perform the expected relevant functions mentioned above. In this regard, Schjenken and Robertson (1) indicated that seminal bioactive signaling factors, including cytokines, would circulate in semen as (1) free soluble in SP, (2) bound to spermatozoa, or (3) encapsulated within extracellular vesicles (EVs). The EVs are lipid membrane nano-size vesicles released by most functional body cells that play a key role in cell-to-cell communication (24). Accordingly, the present study aimed to examine the source of TGF-β isoforms, their release and transport mode in semen, with particular emphasis on the role played by seminal EVs (sEVs). The study has been carried out in porcine, a species of great economic interest and considered an excellent animal biomodel for human medicine (25, 26). Another reason for the choice of the porcine species was that TGF-β isoforms are found in biologically active form in SP (5), with a functional half-life as short as 2–3 min (27), which may hinder their functional performance in the female genital tract after mating or artificial insemination (AI).

The EVs carry active biomolecules, including cytokines, which are transferred from source to target cells, promoting them toward specific functional responses (28–30). Pig SP contains large amounts of sEVs (31, 32), whose cargo and functional role are still poorly investigated (33). Accumulating evidence indicate that EVs would have immunoregulatory properties (34), including those isolated from human and porcine SP (35–40). In porcine Bai et al. (35) reported that sEVs induce immune-related genes expression in the endometrium, a response that could be mediated by immunoregulatory molecules loaded in sEVs. The present study aimed to assess, for the first time in any mammalian species, whether sEVs carry any of the three isoforms of TGF-β. The TGF-β isoforms are among the cytokines found in cancer cell-derived EVs (29, 30) and they were found either inside, as cargo, and outside, membrane-bound, of EVs (41). Accordingly, this study also aimed to evaluate whether TGF-β isoforms were inside and/or outside of sEVs.

To achieve the proposed goals, the present experimental study included immunohistochemistry (IHC) analysis of tissues of male reproductive tract, immunocytochemistry (ICC) and imaging flow cytometry analysis of ejaculated spermatozoa, isolation and characterization of sEVs, and measurement of the three TGF-β isoforms in SP and sEVs using Luminex xMAP^®^ technology.

## 2. Materials and methods

### 2.1. Animals, samples, and experimental design

Eight Landrace × Large White crossbreed boars belonging to Topigs Norsvin España (Madrid, Spain), were used as donors of ejaculates and internal reproductive tissues. All boars were housed in a Spanish AI-center (Calasparra, Murcia) that fulfills the Spanish (ES300130640127; August 2006) and European (ES13RS04P; July 2012) guidelines for ejaculate collection, semen AI-doses commercialization and animal welfare. The boars were allocated in individual pens within a climate-controlled environment building (16 h of natural/artificial light and 15–25°C), with free access to water and fed with commercial feedstuff according to the nutritional requirements for adult boars subjected to regular ejaculate collections.

**Figure 1:**
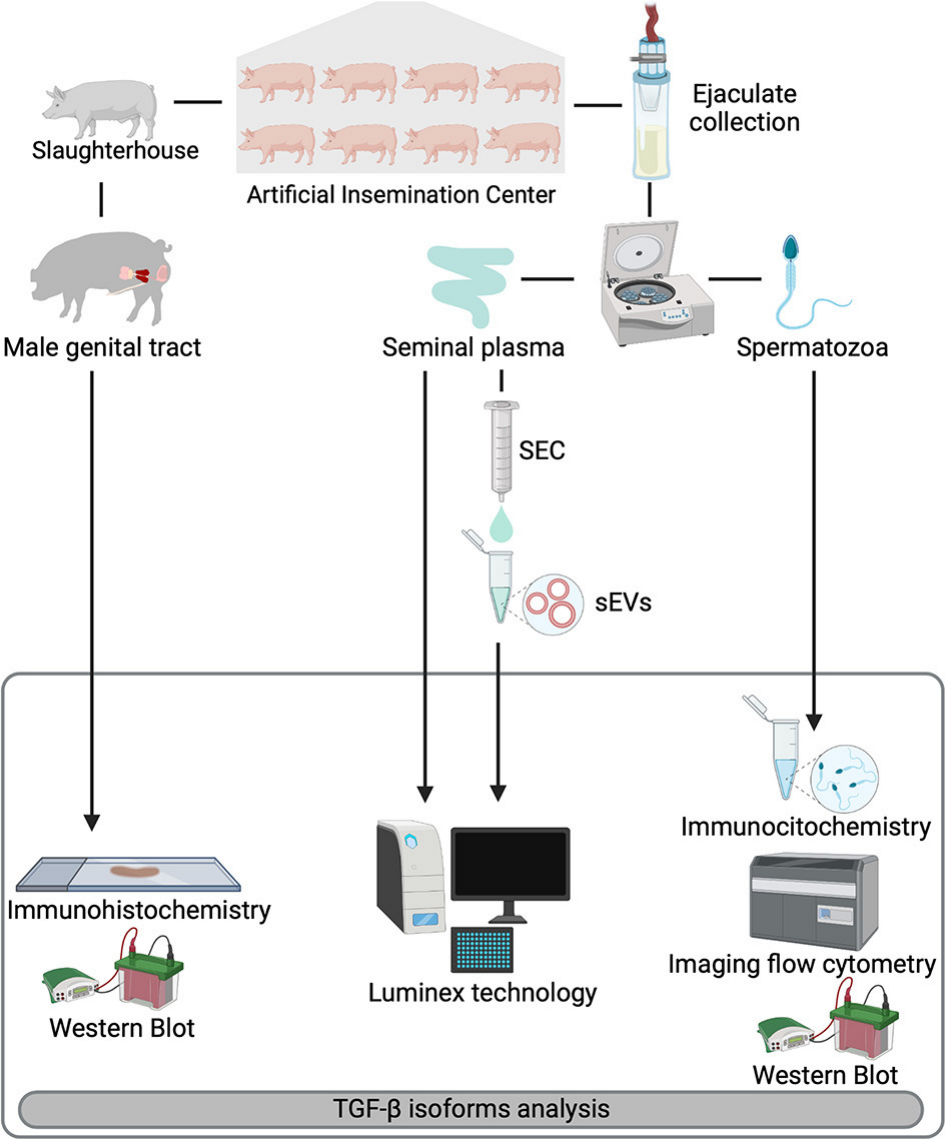
Overview of the experimental design. Entire ejaculates and selected reproductive organs [testis, epididymis, and accessory sex glands (prostate, seminal vesicles, and bulbourethral glands)] were collected from eight healthy breeding male pigs included as semen providers in artificial insemination programs. Ejaculates were centrifuged to separate spermatozoa from seminal plasma (SP). SP samples were subjected to a size-exclusion chromatography (SEC) based protocol for isolation of seminal extracellular vesicles (sEVs). Expression of TGF-β1, -β2, and -β3 were analyzed in reproductive tissues (immunohistochemistry), spermatozoa (immunocytochemistry and imaging flow cytometry) and in SP and sEVs (Luminex xMAP^®^ technology). Presence of TGF-β isoforms in the reproductive tissues and spermatozoa was confirmed by Western blot.

Schematic overview of the experimental design is shown in Figure 1. Entire ejaculates were collected using a semi-automatic collection procedure (Collectis^®^, IMV Technologies, L'Aigle, France) and all ejaculates used in the study fulfilled the standard of sperm quantity and quality thresholds for preparing commercial semen AI-doses (namely >200 × 10^6^ spermatozoa/mL, >70% of them motile and >75% depicting normal morphology). The ejaculates were twice centrifuged at 1,500 × g at room temperature (RT) for 10 min (Rotofix 32A, Hettich Centrifuge UK, Newport Pagnell, Buckinghamshire, England, UK) to separate the sperm pellet from the SP. The SP samples were treated with protease inhibitors (Roche complete™, Protease Inhibitor Cocktail tablets; Basel, Switzerland). Both resulting samples, spermatozoa, and SP, were shipped to the Animal Andrology laboratory of the University of Murcia (Murcia, Spain) in a cooler box (5°C). The boars, still healthy and competent as semen providers, were slaughtered solely for genetic replacement reasons in an industrial slaughterhouse (Matadero de la Mata de los Olmos, Teruel, Spain). The genital tracts of boars were recovered immediately after slaughter and tissues samples from testis, epididymis, and accessory sex glands were collected following the procedures described by Barranco et al. (42) and shipped to the Animal Andrology laboratory of University of Murcia (Spain) in a cooler box (5°C).

At the Animal Andrology laboratory, each sperm pellet sample was split into two aliquots; one stored at −80°C for Western Blot (WB) analysis, and the other processed for ICC analysis as described below. The SP samples were centrifuged (1,500 × g for 10 min at RT; Sorvall™ STR40, Thermo Fisher Scientific, Waltham, MA, USA) to collect SP free from cell debris. The resulting SP samples were stored at −80°C (Ultra Low Freezer; Haier, Schomberg, Ontorio, Canada) for subsequent isolation of sEVs and TGF-βs analysis following the procedures described below. Tissues samples (1 × 1 cm and 1 mm thick) of medial testis, the caput, corpus, and cauda segments of epididymides, and mid-areas of the prostate, the seminal vesicles and the bulbourethral glands were either frozen in liquid nitrogen (for WB analysis) or immersion fixed in Bouin solution for 12 h at RT, immersed in alcohol 70 %, dehydrated, immersed in toluene and embedded in paraffin. Four μm-thick slices were cut and mounted on glass slides for IHC analysis.

### 2.2. Seminal EVs isolation and characterization

Aliquots of 50 μL of each of the 80 SP samples were mixed to generate eight SP pools to avoid confounding boar and sample effects. The sEVs were isolated from each SP pool following the protocol described by Barranco et al. (43). The method combines serial centrifugations, ultrafiltration, and size exclusion chromatography (SEC) and is considered suitable for analyzing cytokines associated with EVs as it minimizes contamination by soluble cytokines (44). Briefly, 4 mL of SP samples were centrifuged (3,200 × g at 4°C for 15 min) and the resultant supernatants were centrifuged again (20,000 × g at 4°C for 30 min). The final resulting supernatants (2 mL) were diluted in 0.22-μm filtered phosphate buffered saline (PBS, Merck, Darmstadt, Germany; 1:2, v:v), filtered (0.22 μm; Millex^®^ Syringe Filters, Merck) and concentrated (Amicon^®^ Ultra-4mL centrifugal filter MWCO 10 kDa; Merck; 3,200 × g at 4°C for 90 min). The resulting samples (~2 mL) were fractionated by SEC. Briefly, 10 mL SEC-columns were handmade using filtration tubes (12 mL; Merck) stacked with Sepharose-CL2B^®^ (Merck) and washed with of 0.22-μm filtered PBS (30 mL). Then, samples were loaded on SEC-column together with 0.22-μm filtered PBS. Twenty sequential 500 μL eluted fractions were collected and the fractions 7 to 10 (enriched in EVs) were selected and mixed. The resulting sEV-samples (2 mL) were ultrafiltered (Amicon^®^ Ultra-2mL centrifugal filter MWCO 100 kDa; Merck; 3,200 × g at 4°C for 90 min) to remove soluble contaminating proteins and to concentrate the sEV-samples into 200 μL. Thereafter, sEV-samples were stored at −80°C (sEV-samples; Ultra Low Freezer; Haier Inc., Qingdao, China) until sEV characterization and measurement of TGF-β isoforms. The sEVs were characterized using several and complementary characterization procedures following International Society for Extracellular Vesicles guidelines (45). Specifically, sEVs were characterized in terms of (1) concentration and size distribution by measuring total protein concentration and using nanoparticle tracking analysis (NTA) and dynamic light scattering analysis (DLS); (2) morphology by cryogenic electron microscopy (Cryo-EM); (3) identification of EV-specific protein markers by flow cytometry; and (4) purity by measuring albumin content by flow cytometry. The details of this EV-characterization are provided in Supplementary File 1.

### 2.3. Immunohistochemistry

Section from the reproductive tissues were deparaffinized with xylene, rehydrated in ethanol (100, 96, and 70%) and distilled water. Endogen peroxidase activity was quenched with 0.3 % H_2_O_2_ at RT for 30 min. The tissue sections were then incubated overnight at 4°C with the TGF-βs antibodies (TGF-β1: ab25121, Abcam, Cambridge, UK; TGF-β2: CPA7447, Cohesion Biosciences, London, UK; TGF-β3: Cat 365274, US biological life, Salem, Massachusetts, USA) diluted 1:50 in PBS containing 1 % of Bovine Serum Albumin (BSA, Merck; PBS/BSA). Then, the sections were incubated with biotinylated goat anti-rabbit IgG secondary antibody (AP-132B, Invitrogen, Waltham, Massachusetts, USA) diluted 1:200 in PBS/BSA at RT for 45 min. Thereafter, the tissue sections were incubated for 30 min with Streptavidin conjugated with Horseradish Peroxidase (HRP, GE Healthcare, Chicago, Illinois, USA) diluted 1:300 in PBS/BSA. Then, for the visualization of peroxidase activity, the tissue sections were incubated in a substrate–chromogen solution containing 0.025 mg/mL 3, 3' diaminobenzidine (DAB, Merck) and 0.015% H_2_O_2_ until a brown color was visible. Counterstaining was carried out using Hansen's hematoxylin stain for 5 s. Finally, the tissue sections were dehydrated with a gradually increasing concentration of ethanol (70, 96, and 100 %), washed in xylene and mounted with DPX (Prolabo^®^, Hermosilla, Sonora, Mexico). The tissue sections were examined with an Olympus BX-51 light microscope (Olympus Co., Tokyo, Japan) and photographs were acquired with an Olympus DP-25 digital camera connected to the microscope. Kidney (pig), amniotic membrane (human) and spleen (pig) tissue sections were used for positive and negative (without primary antibody) controls for TGF-β1, -β2, and -β3, respectively. Representative tissue images of these negative controls are provided in Supplementary Figure 1.

### 2.4. Western blot

Frozen reproductive tissues and spermatozoa were thawed on ice and proteins were extracted by homogenization and incubated 60 min on ice with extraction buffer [PBS with sodium dodecyl sulfate (SDS) at 1% (Merck)] supplemented with protease inhibitors (Complete Mini EDTA-free; Roche, Mannhein, Germany). Protein quantification was performed using the BCA protein Assay Kit (Thermo Fisher Scientific, Waltham, Massachusetts, USA). The precipitation of proteins was performed with acetone to eliminate the effect of eventual interfering substances. The protein suspension was denatured in loading buffer by heating them to 95°C for 5 min and 20 μg samples were loaded into a Mini-PROTEAN TGX precast gels 4–15% Bis-Tris SDS-PAGE gel (Bio-Rad Laboratories, Hercules, CA, USA). Electrophoresis was run at 180 V for 40 min and performed with protein standards Precision Plus Protein Dual Color Standards (Bio-Rad Laboratories). The proteins were transferred to an Immobilon-P membrane (Merck) by semidry electrophoretic transfer at 120 mA/membrane for 60 min (TGF-β1 and TGF-β2) or at 150 mA/membrane for 45 min (TGF-β3) and blocked overnight at 4°C in TRIS-buffered saline (TBS)-Tween-20 at 0.2% supplemented with 5% dry milk non-fat (TBS-Tm). Thereafter, washed membranes (three times of 5 min each one in TBS-Tm) were incubated at 4°C overnight with a primary antibody against TGF-β1, -β2, and -β3 (TGF-β1: ab25121, Abcam; TGF-β2: CPA7447, Cohesion Biosciences; TGF-β3: Cat 365274, US biological life) diluted 1:500 in TBS-Tm. Then, the membranes were washed again and incubated for 90 min with biotinylated goat anti-rabbit IgG secondary antibody (AP-132B, Invitrogen) diluted 1:3,500 in TBS-Tm. Then, the membranes were incubated 45 min with HRP Streptavidin (GE Healthcare, UK) diluted 1:3,500 in TBS-Tm. The immunoreactive bands were located with Clarity Western ECL Substrate (Bio-Rad Laboratories). The images of the blotting were obtained using the Amersham^TM^ Imager 600 (GE Healthcare Europe, GmbH, Freiburg, Germany) and densitometry was performed using Image J software (http://rsb.info.nih.gov/ij/index.html). Ponceau was used for total protein normalization (46). Kidney (pig), amniotic membrane (human) and spleen (pig) tissues were used as positive controls for the mature form (~15 kDa) of TGF-β1, -β2, and -β3, respectively.

### 2.5. Sperm immunocytochemistry (ICC) and imaging flow cytometry

Sperm pellets were diluted (2 x 10^6^ sperm/mL in PBS supplemented with 10% of fetal calf serum (PBS-FCS; Merck) and 100 μL of the resulting sperm samples were incubated with 15 μL of 4',6-diamidino-2'-phenylindole dye (DAPI, 5 μg/mL; Merck) in the dark at RT for 15 min. Thereafter, the sperm samples were centrifuged (400 × g for 5 min) and the resulting sperm pellets were incubated in the dark at RT for 30 min with primary antibodies against TGF-βs (TGF-β1: ab25121; TGF-β2: ab113670 and TGF-β3: ab227711, Abcam; 5 μg/mL). The sperm samples were fixed with paraformaldehyde 1 %. Thereafter, sperm samples were washed twice (diluted in PBS-FCS and centrifuged at 400 × g during 5 min). The resulting sperm pellets were extended in 500 μL of biotinylated goat anti-rabbit IgG secondary antibody (AP-132B, Invitrogen) diluted 1:200 and incubated in darkness at RT for 30 min. Thereafter, sperm samples were again washed, and the resulting sperm pellets were extended in 500 μL of PBS-BSA 3 % plus 1.25 μL of Alexa Fluor 555-conjugated streptavidin (S-32355, 1:400; Thermo Fisher Scientific) and incubated in darkness at RT for 20 min. Finally, the sperm suspensions were washed, and the resulting sperm pellets were extended in 500 μL of PBS-BSA 3% for flow cytometry analysis. Sperm samples without primary antibody were used as negative control.

Image-based flow cytometry sperm analysis was performed with an ImageStreamX MkII (ISX MKII, Amnis, Luminex Corporation, Austin, TX, USA) equipped with five lasers (120 mW 405 nm, 200 mW 488 nm, 200 mW 561 nm, 150 mW 642 nm, 70 mW 785 nm (Side Scatter, SSC). Sample acquisition was made using the INSPIRE data acquisition software (version 200.1.388.0, Amnis, Luminex Corporation). The lasers were set to a power of 100 mW 405 nm, 100 mW 488 nm, 100 mW 561 nm, 438 mW 785 nm (SSC) and data were acquired using 60X objective with a 7 μm core size providing a pixel size of 0.3 μm^2^. The flow rates were set to low flow speed/high sensitivity. The DAPI was excited by a 100 mW 405 laser and detected on channel 7 (430–505 nm filter). The Alexa 555 was excited by a 100 mW 561 nm laser and detected on channel 3 (560–595 nm filter). Channels 1 (430–470 nm filter) and 9 (575–595 nm filter) were used as brightfield channels, and channel 6 (745–800 nm filter) for SSC detection. A minimum of 10,000 events were acquired over three biological replicates for each TGF-β isoform and they were analyzed using the Image Data Exploration and Analysis Software (IDEAS^®^ version 6.2.187.0; Amnis, Luminex Corporation). Spermatozoa were grouped according to DAPI labeling as showing intact (negative DAPI) or damaged plasma membrane (positive DAPI). Sperm that expressed TGF-βs showed red fluorescence. The fluorescence intensity was analyzed using an ISX MKII image flow cytometer, quantified using the IDEAS^®^ software and expressed in arbitrary units.

### 2.6. Measurement of TGF-βs concentration

The concentrations of TGF-β1, -β2 and -β3 were measured in SP and sEVs samples using Luminex xMAP^®^ technology and the TGF-β1,2,3 Magnetic Bead Kit for 96-well plate assay (Cat#TGFB-64K-03 for pig, human, mouse, rat, non-human primate, canine, feline reactivity; Merck) following the protocol recommended by the manufacturer. After thawing at RT and before analysis, the SP as well as lysed (1 % of sodium dodecyl sulfate and 0.1% of Triton X-100 in PBS; Merck) and non-lysed sEV-samples were acidified (pH < 3) with 5 μL of 1N HCl and extended (SP: 1:30, v:v; sEV: 1:5, v:v) with the sample diluent provided in the kit. A standard six-point curve was built for each TGF-β isoform and samples (SP and sEVs). The serum matrix and the two controls provided in the kit were used to ensure precision in the measurements. Briefly, 20 μL of pre-treated SP and lysed and non-lysed sEV-samples were diluted with the sonicated bead solution and incubated at 4°C in the dark overnight. Thereafter, the detection antibody was added and incubated at RT in the dark for 60 min. Then, streptavidin-phycoerythrin was added and incubated at RT for 30 min. Plate reading was performed on a MAGPIX^®^ (Luminex Corporation) using xPONENT software version 4.2 (Luminex Corporation) and MILLIPLEX^®^ Analyst Version 5.1 (Merck) for acquisition and analysis of the data, respectively. A minimum of two technical replicates was analyzed for each sample. The final concentration, recorded as pg/mL, was the mean of the technical replicates. A single standard curve created from the standard curve of each plate was used to normalize the data from different plates analyzed. The intra-assay and inter-assay variability were < 10 and 15%, respectively. The intraclass correlation coefficients of the technical replicates were >0.8.

### 2.6. Statistical analysis

Quantitative data were statistically analyzed using Prism software (version 9.4.1; GraphPad Software, Inc., San Diego, CA, USA). First, the normal distribution of data was analyzed by the Shapiro-Wilk normality test. The WB densitometry data of each TGF-β isoform were normalized with the data of corresponding positive control and analyzed by one-way ANOVA using Tukey's test for multiple comparisons. Data for the percentages of sperm expressing TGF-βs were analyzed by the chi-square test and those for fluorescence intensity by the Mann-Whitney test (differences between sperm with damaged and intact membranes) and the Kruskal-Wallis test (differences between the three isoforms of TGF-βs). Data of TGF-β isoforms in SP and sEVs were analyzed by the Mann-Whitney test (differences between SP and sEVs and between inside and outside of sEVs). The relationship between free TGFβs in SP and those carried by sEVs was measured using Pearson's correlation coefficient. Results were expressed as mean SD, unless otherwise stated.

## 3. Results

### 3.1. The three TGF-β isoforms are expressed in epithelial apical blebs along the male reproductive tract

**Figure 2:**
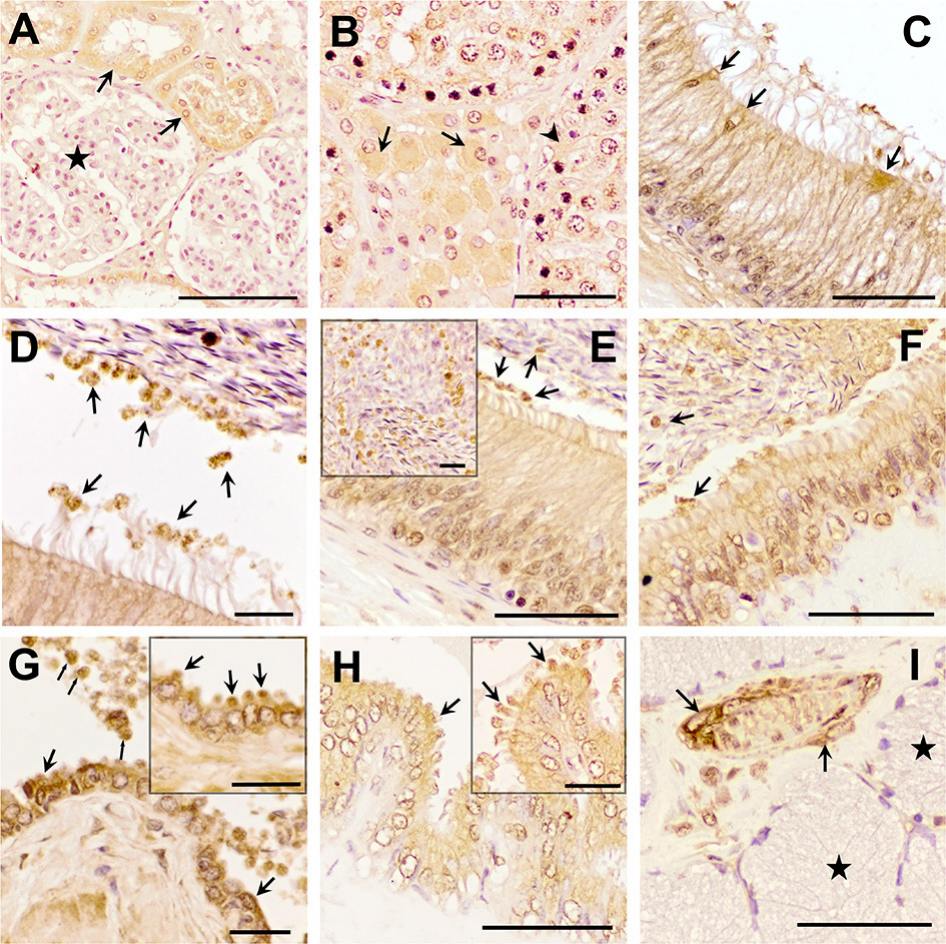
Immunohistochemistry of Transforming Growth Factor (TGF)-β1 in pig male reproductive tissues. Immunolabelling in **(A)** pig kidney (positive control), present in the cytoplasm of the cells of the distal tubules (arrows) but not in renal corpuscles (stars); **(B)** testes, in the cytoplasm of some of the Leydig cells (arrows) and Sertoli cells (arrowhead); **(C, D)** caput epididymis, in clear cells and in large and small vesicular structures; **(E, F)** corpus and cauda epididymis, in vesicular structures close to the stereocilia of principal cells and to spermatozoa (arrows); **(G)** prostate, in luminal vesicles (thin arrows) and in the cytoplasm of the epithelial cells (in detail their apical blebs, thick arrows); **(H)** seminal vesicle, in apical blebs of epithelial cells (arrows); **(I)** bulbourethral gland, in vascular smooth muscle (arrows), but not in secretory epithelium (stars). Scale bars, **(A–C, E, F, H, I)** 50 μm; **(D, G)** 20 μm; and **(E, G, H)** detail 20 μm.

**Figure 3:**
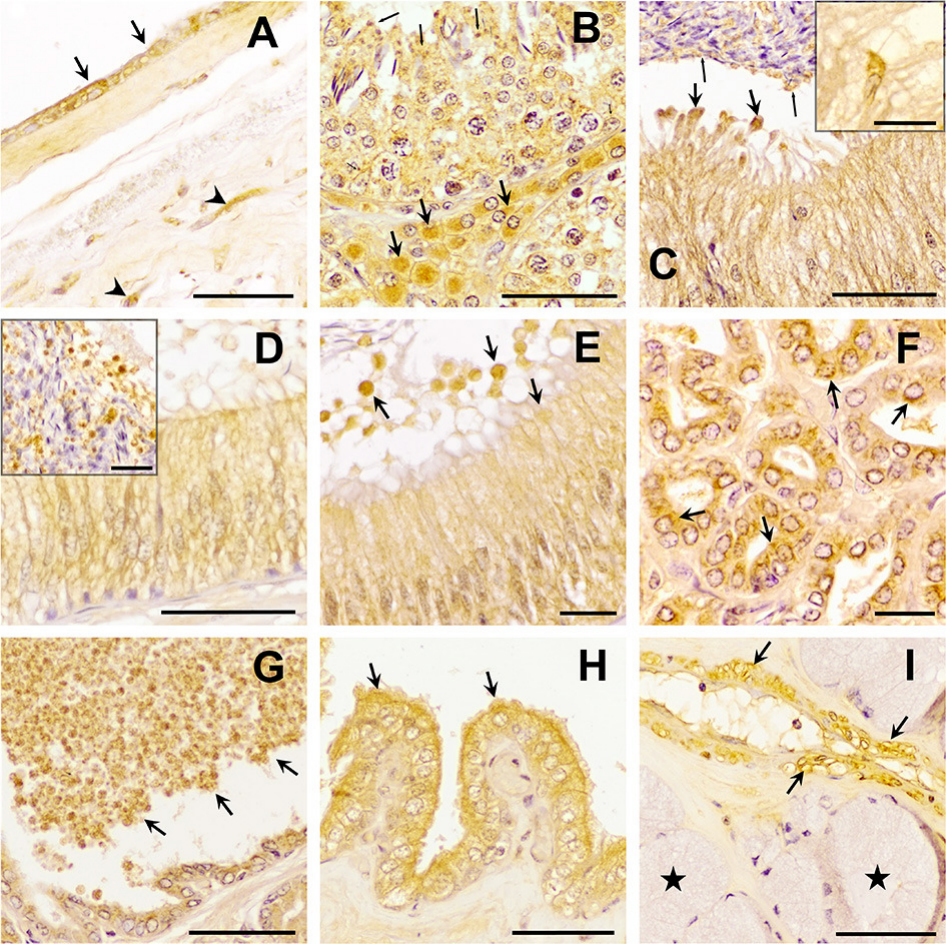
Immunohistochemistry of Transforming Growth Factor (TGF)-β2 in pig male reproductive tissues. Immunoreactivity in **(A)** human amniotic membrane (positive control), present in epithelial (arrows) and mesenchymal (arrowhead) cells; **(B)** testes, in the cytoplasm of Sertoli cells (arrowhead) and Leydig cells (arrows) and in the residual bodies of the spermatids (thin arrows); **(C)** caput, **(D)** corpus, and **(E)** cauda epididymis, in the cytoplasm of the principal cells and in luminal vesicles that, secreted from principal cells [thick arrows, section **(D)**], contained smaller vesicles inside, close to spermatozoa [section **(C)** thin arrows]; **(F, G)** prostate, in the cytoplasm of epithelial cells [section **(F)**, arrows] and luminal vesicles [section **(G)** arrows]; **(H)** seminal vesicle, in the cytoplasm and apical blebs (arrows) of epithelial cells; **(I)** bulbourethral gland, in vascular smooth muscle (arrows), but not in secretory epithelium (stars). Scale bars **(A–D, G–I)** 50 μm; **(E, F)** 20 μm; [**(C, D)** details] 20 μm.

**Figure 4:**
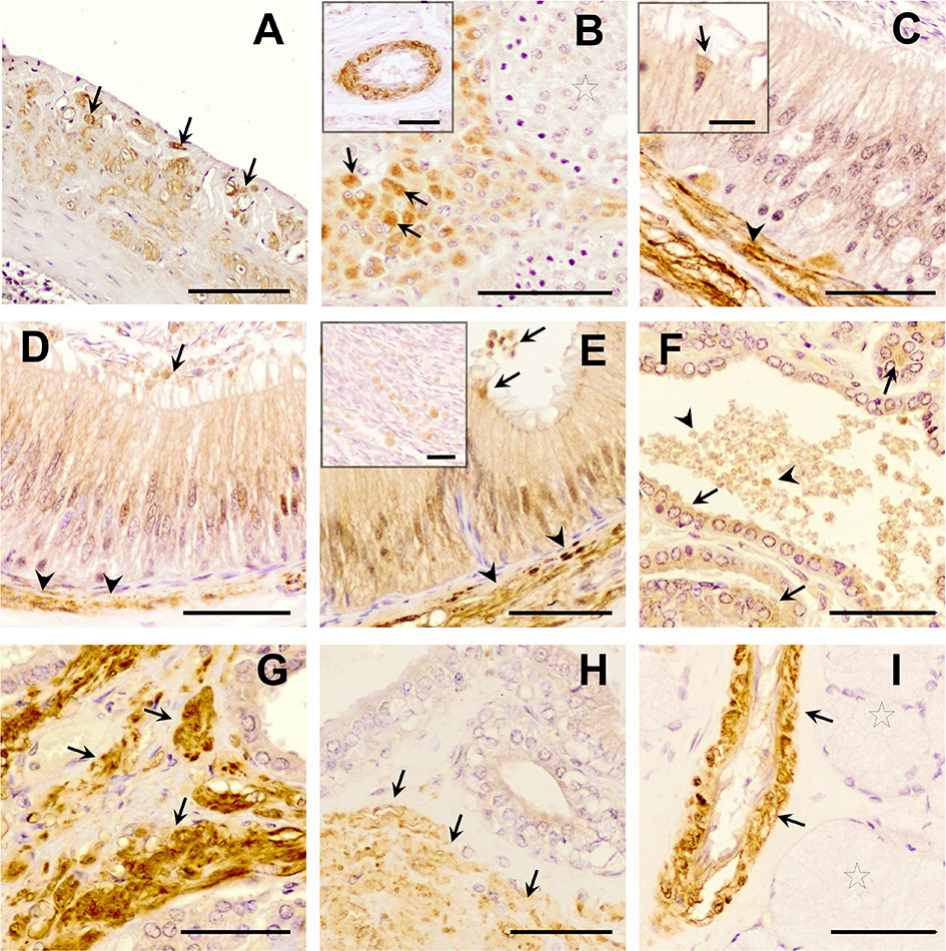
Immunohistochemistry of Transforming Growth Factor (TGF)-β3 in pig male reproductive tissues. Immunolabelling in **(A)** pig spleen (positive control), present in the trabeculae and capsule; **(B)** testes, in the cytoplasm of Leydig cells (arrows) and in vascular smooth muscle (in detail); **(C)** caput, **(D)** corpus, and **(E)** cauda epididymis, in smooth muscle cells (arrowheads), in the cytoplasm of the clear cells [detail in section **(C)**, arrows], and a weak immunostaining in the cytoplasm of principal cells and in luminal vesicles (arrows); **(F, G)** prostate, in epithelial cells [arrows, section **(F)**], in luminal vesicles [arrowhead, section **(F)**] and in smooth muscle cells [arrows, section **(G)**]; **(H)** seminal vesicle, in the smooth muscle cells (arrow) and the epithelium; **(I)** bulbourethral gland in vascular smooth muscle cells (arrows), but not in secretory epithelium (stars). Scale bars, **(A)** 100 μm; **(B–I)** 50 μm; [**(B)** detail] 50 μm; and [**(C, E)** detail] 20 μm.

The three TGF-β isoforms were expressed in testis, epididymis, and accessory sex glands (Figures 2–**4**). In testis, TGF-β1 and -β2 were weakly expressed in the seminiferous epithelium, specifically in Sertoli cells and immature sperm forms. The three TGF-β isoforms were expressed in the interstitium, particularly in the cytoplasm of some Leydig cells (Figures 2B, 3B, 4B). In the epididymis, the three TGF-β isoforms were expressed, albeit weakly, in the supranuclear cytoplasm of the principal cells of the epithelium of the caput, corpus and cauda (Figures 2C–F, 3C–E, 4C–E). In addition, TGF-β1 and -β2 were also expressed in the cytoplasm of epithelial clear cells of the caput (Figures 2C, 3C, 4C). The three TGF-β isoforms were also expressed in apical blebs of epithelial cells and in large vesicular structures around the stereocilia. These vesicular structures contained other small ones that also expressed TGF-β1, -β2, and -β3. Both large and small vesicular structures were also found in epidydimal lumen surrounding maturing spermatozoa (Figures 2C–F, 3C–E, 4C–E). The smooth muscle layer of epididymis also showed a marked immunostaining for TGF-β3 (Figures 4C–E).

In the accessory sex glands, the three TGF-β isoforms were expressed in the prostate, seminal vesicles, and bulbourethral glands. In the prostate were expressed in the principal epithelial cells, specifically in the supranuclear cytoplasm and in the apical edge, and in intralumenal vesicular structures (Figures 2G, 3F, G, 4F, G). TGF-β3 was also expressed in the muscle cells of the interstitium. In the seminal vesicles, the glandular epithelium exhibited high expression for TGF-β1, -β2, but weak for -β3, particularly the apical membrane of the epithelium and apical blebs of the membrane of secretory cells (Figures 2H, 3H, 4H). TGF-β3 was also expressed in muscle cells. In the bulbourethral glands, the three TGF-β isoforms were only expressed in the smooth muscle surrounding the arterioles (Figures 2I, 3I, 4I).

**Figure 5:**
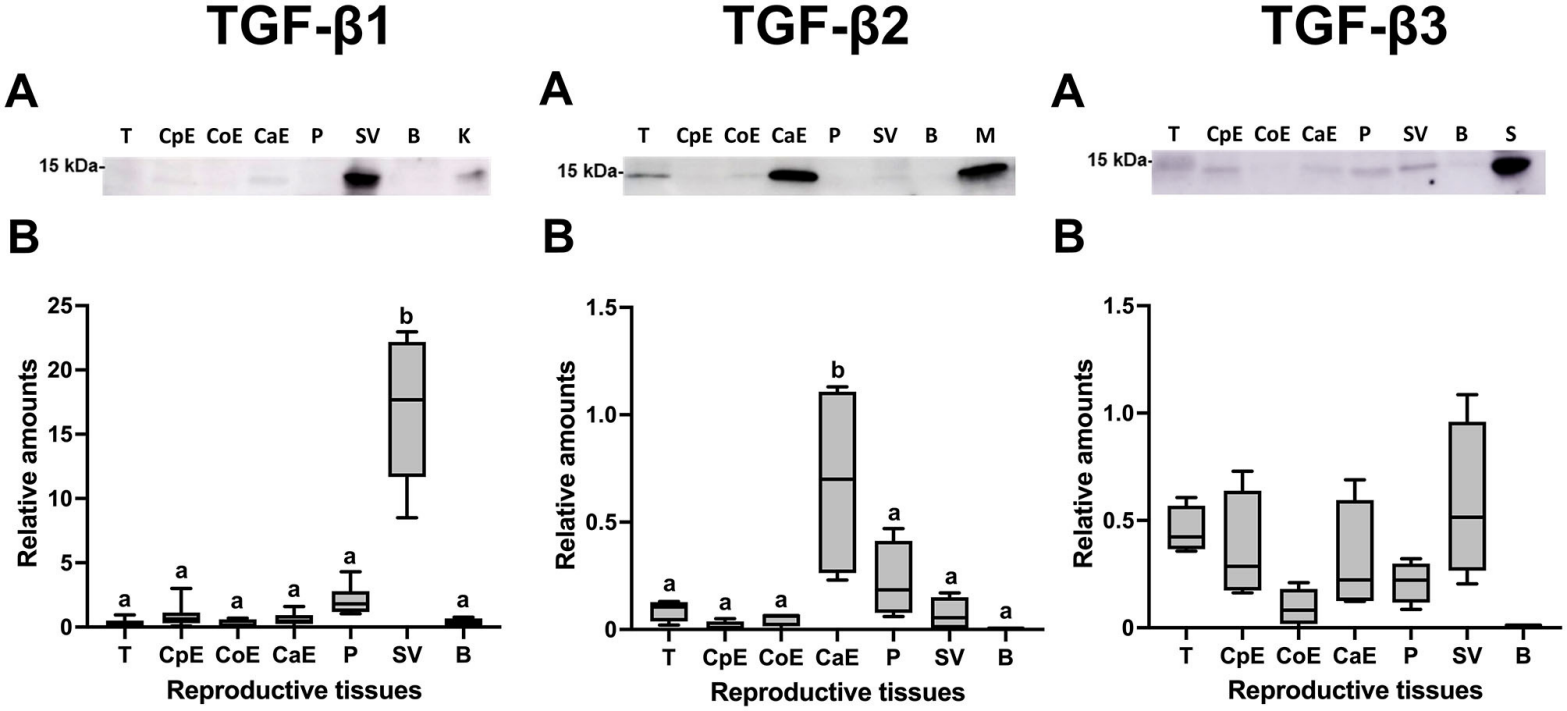
Western blot analysis and relative amount of Transforming Growth Factor (TGF)-β1, -β2 and -β3 in pig male reproductive tissues. **(A)** TGF-β1, -β2, and -β3 expression of mature band (~15 kDa) in internal reproductive tissues: testes (T), caput epididymis (CpE), corpus epididymis (CoE), cauda epididymis (CaE), prostate (P), seminal vesicle (SV) and bulbourethral gland (B). **(B)** Relative amount of mature TGF-β1, -β2, and -β3 band (~15 kDa) in internal reproductive tissues. The plots show data from at least four biological replicates. Boxes enclose the 25th and 75th percentiles, whiskers extend to the 5th and 95th percentiles and the line indicates the median. Different letters above bars indicate significantly different values (*p* < 0.05).

Western blot analysis confirmed the presence of the mature form of TGF-β1, -β2, and -β3 with a molecular weight of ~15 kDa in the tissues of male reproductive organs (Figure 5A). Densitometry showed differences (*P* < 0.05) in the relative amounts of TGF-β1 and TGF-β2 among the male reproductive tissues. Comparatively, TGF-β1 showed the highest relative amounts being highest (*P* < 0.05) in seminal vesicles (Figure 5B). The relative amount of TGF-β2 was highest (*P* < 0.05) in the cauda epididymis (Figure 5B); while the relative amount of TGF-β3 was similar in all male reproductive tissues (Figure 5B).

### 3.2. Ejaculated spermatozoa carry all the three TGF-β isoforms

**Figure 6:**
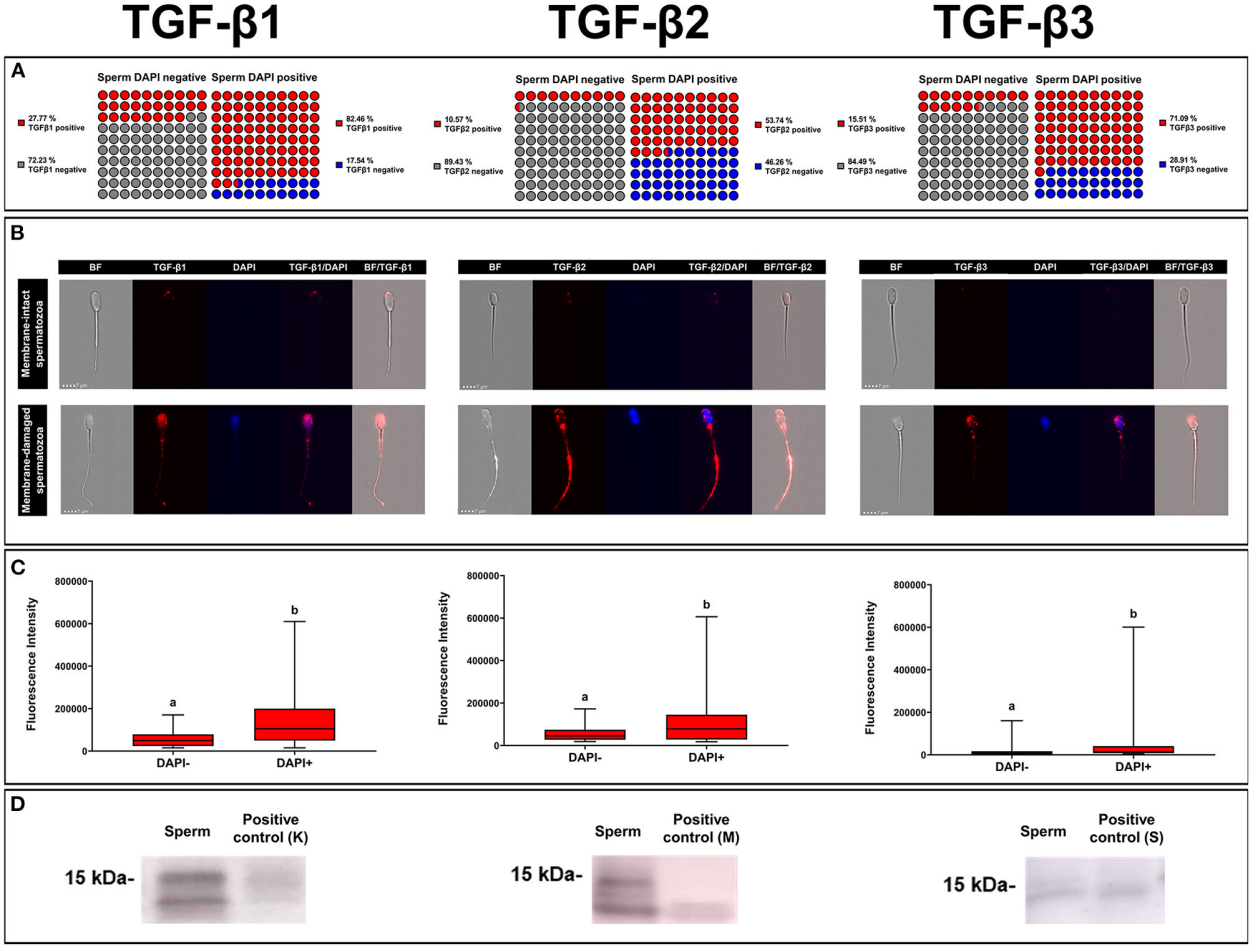
Immunocytochemistry expression of TGF-β1, -β2, -β3 in pig ejaculated spermatozoa. **(A)** Percentage of sperm expressing TGF-β1, -β2, and -β3 in spermatozoa showing intact [4',6-diamidino-2-fenilindol (DAPI) negative] or damaged (DAPI positive) membranes. **(B)** Representative images of TGF-β1, -β2, and -β3 expression (red fluorescence) in spermatozoa with intact (DAPI negative) or damaged (blue fluorescence, DAPI) plasma membrane (bright field, BF). **(C)** Fluorescence intensity of TGF-β1, -β2, and -β3 expression measured in arbitrary units. The plots show data from three biological replicates with more than 10,000 events analyzed per replicate. Boxes enclose the 25th and 75th percentiles, whiskers extend to the 5th and 95th percentiles and the line indicates the median. Different letters above the bars indicate significantly different values (*p* < 0.001). **(D)** TGF-β1, -β2, and -β3 expression of mature band (~15 kDa) in sperm and positive controls [pig kidney (K) for TGF-β1, human amniotic membrane (M) for TGF-β2 and pig spleen tissue (S) for TGF-β3].

A total of 35,290 ejaculated spermatozoa were analyzed with ICC and imaging flow cytometry, specifically 13,724 for TGF-β1, 10,411 for TGF-β2, and 11,155 for TGF-β3. The mean percentages of membrane-intact (DAPI negative) and membrane-damaged (DAPI positive) spermatozoa were 83.47 ± 0.36 and 16.53 ± 0.36 %, respectively, with no difference among TGF-β isoforms. The percentage of spermatozoa expressing any of the three TGF-β isoforms was higher (*P* < 0.001) in those with damaged membrane (DAPI positive) than in those with intact membrane (DAPI negative) (Figure 6A). The percentage of sperm expressing TGF-β1 and TGF-β3 was higher (*P* < 0.001) than that of TGF-β2, irrespective of sperm membrane intactness (Figure 6A). Sperm showed fluorescence in the three domains, i.e., head, neck, and tail (middle and end piece), irrespective of sperm membrane intactness and TGF-β isoform (Figure 6B). Sperm with damaged membrane showed higher (*P* < 0.001) fluorescence intensity than those with intact membrane, irrespective of the TGF-β isoform. The fluorescent intensity was higher for TGF-β1 and lower for TGF-β3 (*P* < 0.001), irrespective of sperm membrane intactness (Figure 6C). Fluorescence was shown mainly in a scattered form in spermatozoa with damaged membrane, and predominantly as small individual spots in spermatozoa with intact membrane, irrespective of the TGF-β isoform (Figure 6B). WB confirmed the presence of the ~15 kDa mature form of TGF-β1, -β2, and -β3 in ejaculated spermatozoa (Figure 6D).

### 3.3. The sEVs carry the three TGF-β isoforms both inside and outside

Seminal plasma contained the three TGF-β isoforms but in highly variable concentrations, showing TGF-β2 and TGF-β3 the highest and lowest concentrations, respectively (Table 1). The SP concentrations of TGF-β1, -β2, and -β3 differed (*P* < 0.0001) among boars and even among ejaculates within the same boars. While TGF-β1 and TGF-β2 were found in measurable concentrations in all the SP samples analyzed, TGF-β3 was not detected in some of them.

**Table 1:** Concentrations (pg/mL) of transforming growth factor beta (TGF-β) isoforms (β-1, -β2, and -β3) in seminal plasma samples from eight healthy breeding male pigs (*n* = 80 semen samples, 10 samples per male) used in commercial artificial insemination programs.

	**TGF-β1**	**TGF-β2**	**TGF-β3**
N° samples	80	80	80
Median	784.07	25360.50	34.50
25th percentile	452.40	14311.34	0
75th percentile	1340.17	41536.27	118.87

**Figure 7:**
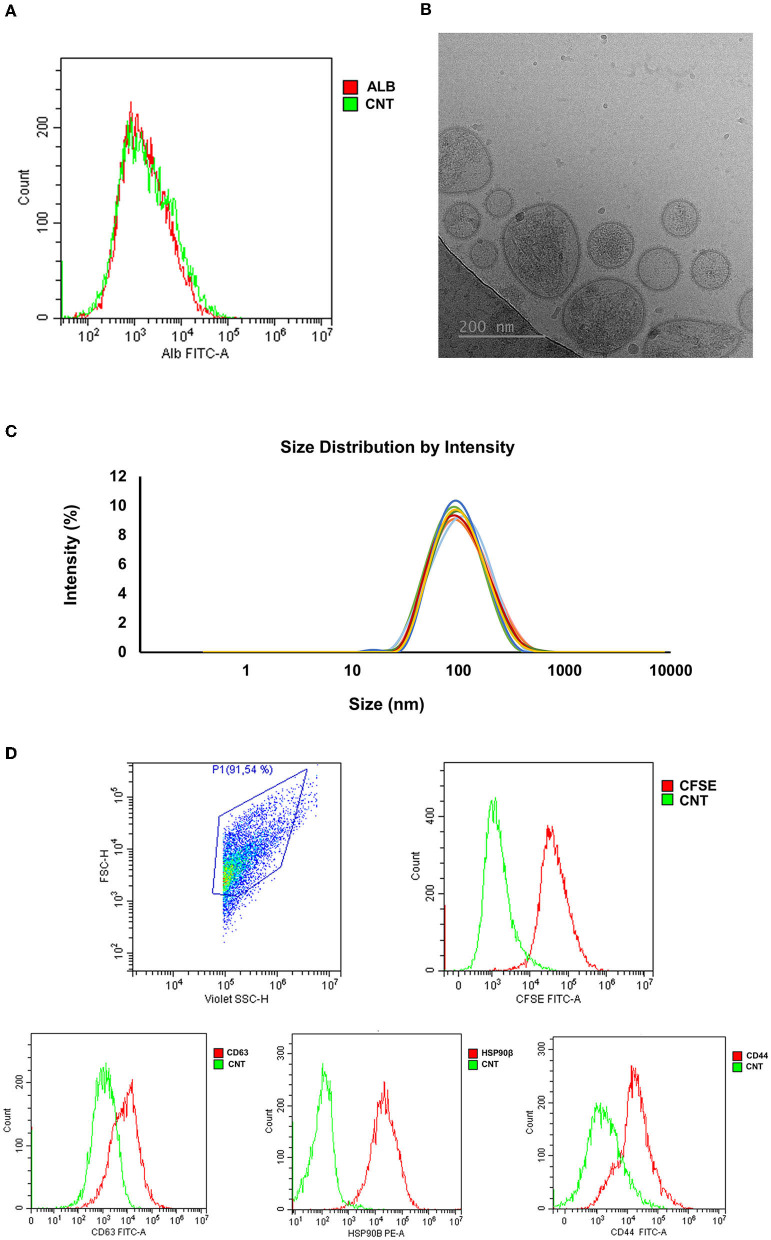
Characterization of seminal extracellular vesicles (sEVs) isolated from pig seminal plasma (SP) (*n* = 8; each sample pooled 10 individual SP samples) by serial centrifugations, size exclusion chromatography and ultrafiltration. **(A)** Representative histogram of albumin (ALB) assessed in EV-samples by flow cytometry. **(B)** Representative image of sEVs assessed by cryo-electron microscopy. **(C)** Particle size distribution of sEVs assessed by dynamic light scattering. Each lines represent the intensity size distribution in each EV-sample. **(D)** Representative histograms of CFSE/CD63/HSP90β/CD44 expression in seminal EVs assessed by flow cytometry. CFSE, Carboxyfluorescein succinimidyl ester; HSP90β, Heat Shock Protein 90β; CNT, Control.

The concentration of sEVs was 11.78 x 10^11^ ± 1.69 x 10^11^ per mL of SP. The sEV-samples displayed a high purity degree as demonstrated by the presence of only 4.21 ± 1.83 % of albumin (Figure 7A). The concentration of total protein in sEV-samples was 211.70 ± 29.27 μg/mL. Cryo-electron microscopy imaging revealed the presence of membrane-intact EVs that showed heterogeneous shapes and were mostly < 200 nm in size (Figure 7B). The size distribution was further confirmed by DLS analysis (EV-diameter; median, 25–75th percentiles: 118.90, 112.11–122.61 nm) (Figure 7C). Protein EV-specific markers of was performed on the base of (1) their physical characteristics in the Forward Scatter and Violet-SSC-A and (2) further discriminated by Carboxyfluorescein succinimidyl ester (CFSE)-labeling, which allowed differentiate intact EVs from both non-EV structures and electronic noise. The percentage of CFSE-positive events was 93.95 ± 5.10 %; and the 81.7 ± 3.42 %, 90.33 ± 5.34 %, and 98.85 ± 0.50 % of them were positive to CD63, HSP90β, and CD44, respectively (Figure 7D).

**Figure 8:**
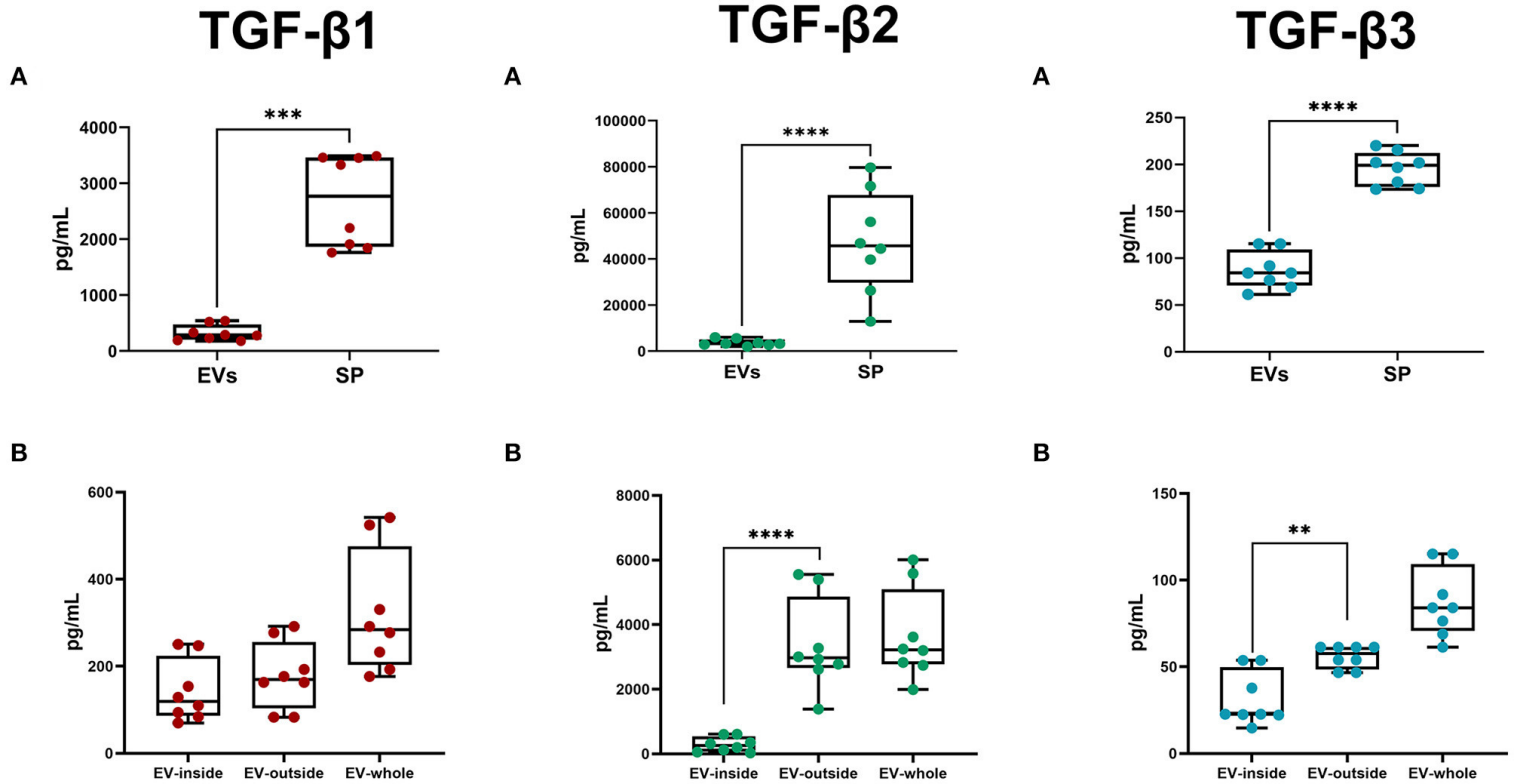
Box-whisker plots showing the concentrations (pg/mL; mean ± SEM) of Transforming Growth Factor (TGF) -β1, -β2, and -β3 in pig. **(A)** Seminal plasma (SP) and seminal extracellular vesicles (EVs; lysed). **(B)** EV-inside (Difference between lysed and non-lysed EVs), EV-outside (non-lysed EVs), and EV-whole (lysed EVs). Data of eight SP pools. Boxes enclose the 25th and 75th percentiles, whiskers extend to the 5th and 95th percentiles and the line indicates the median, and dots represent the seminal TGF-βs values. ****P < 0.00001; ***P < 0.0001; **P < 0.001.

The Luminex analysis revealed that sEVs carried all three TGF-β isoforms. The amount of the three isoforms in sEVs was lower (*P* < 0.0001) than that freely circulating in SP (Figure 8A). Specifically, sEVs carried 13.76, 10.24, and 44.80% of the total SP amount of TGF-β1, TGF-β2, and TGF-β3, respectively (Table 2). There was no correlation between the amount of SP-free TGF-β isoforms and those carried by sEVs. TGF-β isoforms carried by sEVs were present both inside (encapsulated EVs) and outside (membrane-bound EVs) (Figure 8B). There was no difference in the amount of TGF-β1 between inside and outside. However, the amount of TGF-β2 and TGF-β3 was higher (*P* < 0.01) outside than inside.

**Table 2:** Concentrations (pg/mL; mean ± SEM) of transforming growth factor (TGF) -β1, -β2 and -β3 in samples of seminal plasma (SP) and isolated seminal extracellular vesicles (EVs) of healthy breeding male pigs used in commercial artificial insemination programs (*n* = 8, each sample pooled 10 individual SP samples).

	**Seminal plasma**	**Seminal EVs**	**Ratio EVs/SP**
TGF-β1	2,682.61 ± 288.21	321.01 ± 49.67	13.76
TGF-β2	47,214.35 ± 7798.61	3,651.90 ± 498.55	10.24
TGF-β3	195.67 ± 6.29	87.11 ± 6.96	44.80

## 4. Discussion

The present study showed that the three TGF-β isoforms were ubiquitously expressed in the tissues of reproductive tract of male breeding pigs. It also demonstrated that a portion of the expressed TGF-β isoforms were secreted into the extracellular milieu *via* epithelial apical blebs, which would be the main source of seminal EVs (47). The fact that tissues of the male reproductive tract express TGF-β isoforms is not a novelty, as it has been reported by different studies over recent years. For instance, in pig testis (22, 48), in murine testis (49), epididymis (50) and accessory sex glands (13, 23), monkey epididymis (51), and human testis (52). However, the present study would be, to our knowledge, the first in mammalian species to demonstrate that porcine testis, epididymis, prostate, and seminal vesicles expressed and secreted to extracellular milieu all three TGF-β isoforms, secretion that in part would occur through apical blebs from epithelial cells. Specifically, apical blebs of the epididymis, prostate, and seminal vesicles expressed TGF-β1 and -β2. Apical blebs are usually present in epithelial cells and are considered a pathway to release EVs (53). In this regard, it is well known that EVs produced by male reproductive tract tissues are mainly released following an apocrine secretory mechanism involving the cytoplasmic protrusion of large apical blebs containing inside several small vesicles (47). Once in the lumen, the large apical blebs, so-called storage vesicles, disintegrate, releasing the small vesicles to the extracellular milieu (54). These released small vesicles would be the EVs present in the male reproductive tract fluids that gather in the ejaculate. Since these released small vesicles also express TGF-βs, it is reasonable to consider that a part of the TGF-βs released from male pig reproductive tissues to the extracellular milieu would be sEVs-associated. This statement is supported by Aiello et al. (29), who considered EVs as another pathway of cytokine secretion from cells to the extracellular milieu. Aiello et al. (29) suggested that the encapsulation of cytokines in EVs would be a way to preserve them from environmental degradation. In this regard, it is appropriate to point out that pig SP is particularly rich in proteolytic enzymes (55).

The TGF-β isoforms synthesized in male reproductive tract reaching the lumen are present in semen, either in spermatozoa or SP, and reach the female reproductive tract during mating or AI. Seminal TGF-β isoforms would play two different functional roles. On one hand, they would be involved in the functional regulation of the reproductive tract itself by means of autocrine/paracrine pathways (51, 56, 57). On the other hand, they would also play an essential role in the inner female genital tract by promoting a favorable immune environment for both sperm transit and embryo development (1, 58).

The results of ICC and imaging flow cytometry showed that pig ejaculated spermatozoa expressed the three TGF-β isoforms. Although TGF-β1 expression in spermatozoa has already been reported in humans (59, 60), the present study appears to be first in reporting the expression of TGF-β2 and TGF-β3 in any mammalian sperm. The origin of TGF-β isoforms in spermatozoa is still unclear. One possibility is that they originate during spermatogenesis. The present study shows that spermatogonia expressed TGF-β1 and -β2, which would agree with Caussanel et al. (22) and Loveland et al. (61) who reported the expression of TGF-βs at some stages of rodent and porcine spermatogenesis, respectively. Alternatively, TGF-β isoforms in spermatozoa could also come from reproductive fluids. TGF-β isoforms secreted by male reproductive organs and released into the fluid accompanying the spermatozoa could bind to immature spermatozoa, during their transit through the caput and body of the epididymis, or to mature spermatozoa, during their storage in the cauda of the epididymis or/and during ejaculation. The present study showed that both spermatozoa with either intact or damaged membranes expressed the three TGF-β isoforms, displaying a higher expression in those with damaged membranes. This higher expression in spermatozoa with damaged membranes would indicate that most of TGF-β isoforms expressed by spermatozoa would be inside the membranes. Therefore, it is reasonable to consider that TGF-β isoforms inside membranes ought to be product of spermatogenesis, since mature spermatozoa are considered transcriptionally and translationally silent cells (62). The fact that the residual spermatid bodies expressed TGF-β2 would support this assumption. Alternatively, it could be that TGF-βs inside membranes came from the outside and penetrated through the gaps in the damaged plasma membrane, but this seems unlikely unless the membrane damage was extensive, which is unusual in ejaculated sperm from healthy breeding boars. Whether these TGF-β isoforms inside membranes play any functional role in mature sperm remains to be elucidated.

The TGF-β isoforms expressed in membrane-intact sperm would be on the sperm membrane, probably bound on its outer side, although an initial internalization can neither be ruled out. Flow cytometry imaging of membrane-intact sperm showed that TGF-β isoforms were mainly expressed as isolated small spots, more or less numerous depending on the spermatozoon. These images would suggest that TGF-β isoforms could be linked to sEVs that would bind to sperm membranes during their transit through the male reproductive conducts. Unfortunately, we have not been able to prove this hypothesis because, to our knowledge, there are no commercial fluorescent probes that allow labeling EVs without also labeling spermatozoa at the same time. Spermatozoa contain the proteins that are usually labeled to identify EVs, e.g., CD9, CD63, and CD81 (63). However, in this regard, it is well-known that sEVs bind to sperm membranes (64) and that pig ejaculated spermatozoa have membrane-bound sEVs, as recently reported by Roca et al. (33) based on transmission electron microscopy imaging. Whether associated with sEVs or free, it seems proven that pig spermatozoa carry active isoforms of TGF-β bound to their plasmalemma. These TGF-β isoforms would have biological relevance, perhaps protecting spermatozoa during their transit in the female genital tract, preventing attack by lymphocytes, and thus facilitating them to reach the sperm reservoirs safely (59). The fact that membrane-bound TGF-β isoforms take time to degrade, remaining biologically active for several days (65), and that small amount of cytokines, e.g., those found in EVs, are enough to play their full functional role (29), would support this statement. It may also be the case that the bound sEVs would remain attached to the sperm membrane and be transported along to the upper oviduct and even be present during fertilization, as it has been proven for specific SP-proteins (66), thus indicating that components of the pig SP can indeed enter the oviduct to act locally on the epithelium or on fertilization events.

The present study confirmed that porcine SP is rich in the three TGF-β isoforms, which is consistent with previous studies (5, 8, 10). TGF-β isoforms in SP can be found in either the latent or the biologically active form depending on the animal species. Thus, while in humans the latent form predominates (67), in pigs it is the biologically active form (5). The biologically active form in body fluids has a short functional half-life of only 2–3 min, as they are rapidly inactivated by natural circulating inhibitors (27). In this regard, it is timely to remind, once again, that the pig SP is rich in proteolytic enzymes (55). A 2–3 min short functional half-life would conflict with the relevant functional roles attributed to seminal TGF-β isoforms once semen reaches the female reproductive tract, which can occur days after ejaculation when AI is used, as is the case in pigs (68). A possible explanation could be that a part of seminal TGF-β isoforms circulate protected from natural circulating inhibitors, e.g., bound to sEVs. As Aiello et al. (29) point out, sEVs are a safe carrier of cytokines, which facilitates the safe delivery of their active forms to remote target cells, e.g., those of the female reproductive tract *via* sEVs. Shelke et al. (69) reported that TGF-β1 bound to EVs gives longer cell signaling over time compared to freely circulating TGF-β1. The present study demonstrated that a substantial part of the three TGF-β isoforms present in SP were bound to sEVs either as cargo inside, or outside in the so-called molecular corona surrounding the EVs (70), which plays a key role in the functional spectrum of EVs (71). Similar results were achieved by Fitzgerald et al. (72) who reported that a substantial portion of several cytokines circulating in human blood plasma and amniotic fluid, TGF-βs among them, were bound to EVs, either inside or outside. Both locations of TGF-β isoforms, inside the EVs and outside in the molecular corona, ensure a long functional half-life for the active forms of TGF-β isoforms, since in both locations they remain protected from environmental degradation preserving signaling competence for days (72). The presence of active forms of TGF-β isoforms for days in sEVs would support the immunoregulatory properties attributed to sEVs in humans (36–39), porcine (35), and murine (73) species. This functional immunoregulatory activity would be mediated by the TGF-β isoforms localized in the molecular corona, which would bind to TGF-β receptors on the surface of EV target cells, triggering the intracellular signaling events that would elicit the immunoregulatory response (74).

In conclusion, the present study demonstrated that tissues from functional organs of the male pig reproductive tract expressed all three TGF-β isoforms (TGF-β1, -β2, and -β3), releasing them into the ductal lumen in soluble form or associated with sEVs. It also showed that porcine ejaculated spermatozoa expressed all three TGF-β isoforms, both inside and outside; the outer one probably associated with membrane-bound EVs. The study also confirmed that SP contains all three TGF-β isoforms and demonstrated that a substantial portion of them would be associated with sEVs. In summary, the study demonstrated that sEVs would be involved in the cellular secretion of the active forms of seminal TGF-β isoforms and in their safe transport from the male to the female reproductive tract, where they should play essential functional roles regulating the immune environment.

## Data availability statement

The original contributions presented in the study are included in the article/Supplementary material, further inquiries can be directed to the corresponding author.

## Ethics statement

The animal study was reviewed and approved by Bioethics Committee of Murcia University (research code: CBE: 367/2020). Written informed consent was obtained from the owners for the participation of their animals in this study.

## Author contributions

Conceptualization: IP, XL, and JR. Data curation: LMP, HR-M, and JR. Formal analysis: IB, JM-H, LMP, and JR. Funding acquisition: HR-M and JR. Investigation: IB, LP, JM-H, and AP. Methodology: IB, LMP, IP, AP, and XL. Supervision: LMP and JR. Visualization: IB, LP, and JM-H. Writing—original draft: IB and LP. Writing—review and editing: IP, HR-M, and JR. All authors contributed to the article and approved the submitted version.

## Supplementary material

Supplementary FIGURE 1 Negative immunohistochemical controls showing non-immunoreactivity. Representative images of tissue samples not incubated with primary antibodies against **(A)** TGF-β1, **(B)** TGF-β2 and **(C)** TGF-β3. Tissues are **(A)** pig kidney (100 μm inset bar); **(B)** human amniotic membrane (50 μm inset bar); **(C)** pig spleen (100 μm inset bar).

Supplementary File 1 Characterization of seminal extracellular vesicles (sEVs) following Minimal Information for Studies of Extracellular Vesicles 2018 (MISEV2018) guidelines.
