# Supplementary figures and images for: Extracellular vesicles would be involved in the release and delivery of seminal TGF-β isoforms in pigs
Source: Front Vet Sci. 2023 Feb 10;10:1102049. doi: 10.3389/fvets.2023.1102049 (PMC9950116; doi:10.3389/fvets.2023.1102049)

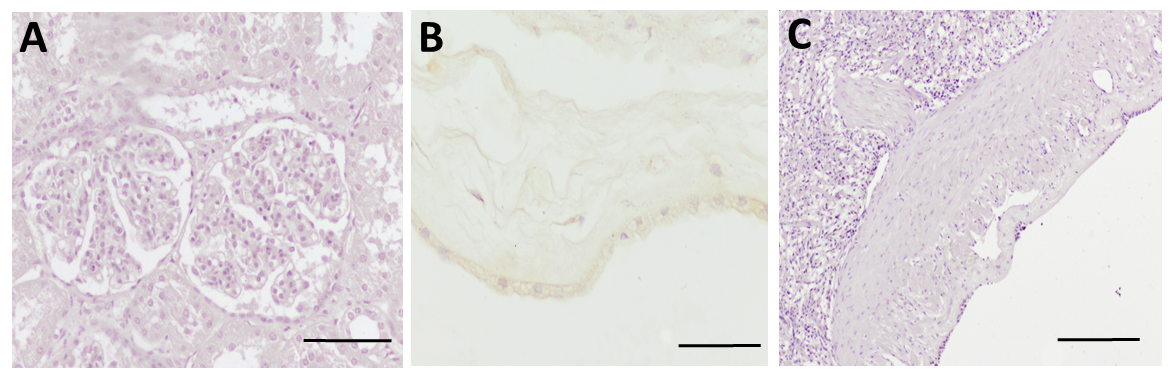

Supplement: Supplementary FIGURE 1 — Negative immunohistochemical controls showing non-immunoreactivity. Representative images of tissue samples not incubated with primary antibodies against (A) TGF-β1, (B) TGF-β2 and (C) TGF-β3. Tissues are (A) pig kidney (100 μm inset bar); (B) human amniotic membrane (50 μm inset bar); (C) pig spleen (100 μm inset bar). [file Image_1.TIF]
