# Supplementary material for: Extracellular vesicles would be involved in the release and delivery of seminal TGF-β isoforms in pigs
Source: Front Vet Sci. 2023 Feb 10;10:1102049. doi: 10.3389/fvets.2023.1102049 (PMC9950116; doi:10.3389/fvets.2023.1102049)
Supplement: Supplementary File 1 — Characterization of seminal extracellular vesicles (sEVs) following Minimal Information for Studies of Extracellular Vesicles 2018 (MISEV2018) guidelines. [file Data_Sheet_1.docx]

Supplementary Material

**Supplementary File 1. Characterization of seminal extracellular vesicles (sEVs) following Minimal information for studies of extracellular vesicles 2018 (MISEV2018) guidelines.**

The total protein content of sEV-samples was assessed using the Micro BCA™ Protein Assay Kit (Thermo Fisher Scientific, Waltham, Massachusetts, USA) and following the manufacturer’s instructions. Prior to the analysis, 25 µL-aliquot of each sEV-sample was treated (1:1, v:v) with a chemical lysis solution (0.1 % Triton and 0.1 % of sodium dodecyl sulphate in purified water; Merck, Darmstadt, Germany) and incubated for 30 min at 37 °C in dark and under shaking. The absorbance was evaluated using a micro-plate reader (PowerWave XS; Bio-Tek Instruments, Winooski, Vermont, USA) at a wavelength of 562 nm.

The particle size distribution of sEVs was assessed by dynamic light scattering (DLS). The analysis was performed using a Zetasizer Nano ZS-system (Malvern Panalytical, Malvern, UK) operating at 633 nm and recording the back scattered light at 173°. For this, a 50 μL-aliquot of each sEV-sample was loaded into a 10 mm pathlength-cuvette. The light scattering was recorded for 150 s. Three measurements were recorded per each sEV-sample. Dispersion Technology Sofware v.5.10 (Malvern Panalytical) was used to converts DLS signal intensity to particle size distribution. The sEV diameter (nm) was calculated based on the peak maximum of the gaussian function.

The morphology of EVs was analyzed by cryogenic electron microscopy (Cryo-EM) following the protocol of Las Heras et al., (2022). Briefly, 3-5 µL aliquot of each sEV-sample was placed on glow-discharged lacey grids that were immersed in liquid ethane at -180°C (Vibrobot, FEI, Eindhoven, The Netherlands). The grids were then observed with a JEM-2200FS/CR electron microscope (JEOL, Tokyo, Japan) operating at 200 kV and images were acquired with a multiscan CCD camera (Model USC 4000, Gatan Inc., Pleasanton, California, USA).

The concentration and protein composition of sEVs were analyzed by flow cytometry using a high-resolution flow cytometer (CytoFLEX S; Beckman Coulter, Life Sciences Division Headquarters, Indianapolis, USA). Fluorescent nanospheres of 50 and 100 nm diameter (Nanobead Calibration Kit, Bang Laboratories Inc. Drive Fisher, Indiana, USA) were used to verify the accuracy of the flow cytometer for the input and counting of EVs. The analysis was restricted to events with EV-specific size (forward scatter, FSC) and complexity (Violet-side scatter (SSC)-A) features. Samples were analyzed using the low flow setting (5-10 μL/min) acquiring at least 10×10^3^ events per sample. An 10 μL-aliquot of each sEV-sample was incubated with CellTrace™ CFSE (Carboxyfluorescein succinimidyl ester; Thermo Fisher Scientific) in order to identify intact sEVs and differentiate them from non-EV structures, including membrane fragments. The sEVs concentration was calculated from the CFSE positive events and dilution rate. The proteins identified were CD63 and CD44 as proteins of category 1 (Transmembrane or GPI-anchored proteins associated to plasma membrane and/or endosomes); 90 kDa heat shock protein (HSP90β) as a protein of category 2 (Cytosolic proteins recovered in EVs); and albumin as a protein of category 3 (Major components of non-EV co-isolated structures). The antibodies used were anti-HSP90β-PE (ADI-SPA-844PE-050, Enzo Life Sciences, Farmingdale, NY, USA), anti-CD63-FITC (Clone REA1055, Miltenyi Biotec, Bergisch Gladbach, Germany), anti-CD44-FITC (MCA4703F, Bio-Rad, Hercules, California, USA) and anti-Albumin-FITC (CLFAG16140, Cedarlane, Burlington, Canada, USA). Detailed protocols are described in Barranco et al., (2019).

**References**

Barranco, I., Padilla, L., Parrilla, I., Álvarez-Barrientos, A., Pérez-Patiño, C., Peña, F. J., et al. (2019). Extracellular vesicles isolated from porcine seminal plasma exhibit different tetraspanin expression profiles. *Sci. Rep.* 9, 11584. doi:10.1038/S41598-019-48095-3.

Las Heras, K., Royo, F., Garcia-Vallicrosa, C., Igartua, M., Santos-Vizcaino, E., Falcon-Perez, J. M., et al. (2022). Extracellular vesicles from hair follicle-derived mesenchymal stromal cells: isolation, characterization and therapeutic potential for chronic wound healing. *Stem Cell Res. Ther.* 13, 147. doi:10.1186/s13287-022-02824-0.
